# Innowave MTB/RIF/INH facilitates timely and accurate diagnosis of multiple-drug resistant tuberculosis as a near POCT technique: a multicenter prospective on-site performance evaluation study

**DOI:** 10.1186/s12941-025-00822-7

**Published:** 2025-09-26

**Authors:** Fen Wang, Long Cai, Zhongfeng Cui, Guanglu Jiang, Hairong Huang

**Affiliations:** 1 National Clinical Laboratory on Tuberculosis, Beijing Key Laboratory on Drug-Resistant Tuberculosis Research, Beijing Tuberculosis and Thoracic Tumor Institute, Beijing Chest Hospital, Capital Medical University, Beiguan St No. 9, Beijing, 101149 China; 2 Medical Laboratory Center, Hangzhou Red-Cross Hospital, Hangzhou, Zhejiang Province China; 3 Clinical Laboratory, The Sixth People’s Hospital of Zhengzhou, Zhengzhou, Henan Province China

**Keywords:** Tuberculosis, Diagnosis, Drug resistance, Rifampicin, Isoniazid, MDR

## Abstract

**Background:**

Many rifampicin (RIF)-resistant (RR) tuberculosis (TB) patients remain sensitive to isoniazid (INH), which challenges the strategy of using RR as an instant indicator of multiple-drug resistance tuberculosis (MDR-TB). A molecular test capable of concurrently detecting RIF and INH resistance is urgently needed.

**Methods:**

The performance of a novel rapid molecular test, Innowave MTB/RIF/INH (InnowaveDX) was evaluated prospectively in three tertiary hospitals. Its capability of detecting resistance to RIF and INH was assessed.

**Results:**

In 767 pulmonary tuberculosis (PTB) patients, InnowaveDX showed significantly higher sensitivity than the Xpert MTB/RIF assay (Cepheid, USA) (74.97% versus 68.18%; p = 0.003, χ^2^ = 8.664). This difference was particularly notable in culture-negative PTB cases (52.73% versus 41.29%; p = 0.001, χ^2^ = 10.565). Both tests demonstrated high specificity in 286 non-TB patients. The overall consistency in RIF susceptibility prediction between InnowaveDX and the Xpert assay was 97.3% (505/519). InnowaveDX identified 83.05% (98/118) of INH-resistant cases as predicted by phenotypic drug susceptibility testing (pDST) and 95.45% (105/110) by another molecular method (MeltPro, Zeesan, China) for INH resistance detection on isolates. In addition, InnowaveDX showed a 99.35% consistency (154/155) with *katG*, *inhA*, and *ahp*C sequencing on sputum samples. The consistency rate for MDR-TB prediction between InnowaveDX and pDST was 93.25% (332/356). The accuracy of using RR to predict MDR-TB varied between 64.1 and 80.5%, depending on the reference method.

**Conclusion:**

InnowaveDX is an easy, rapid, and sensitive molecular test for PTB diagnosis that can detect INH and RIF resistance within 3 h, facilitating MDR-TB diagnosis on the first day of hospital admission.

## Background

China faces a significant TB burden, ranking third globally in the estimated number of cases [1]. The Chinese National TB Program (NTP) confronts a major challenge with drug resistance (DR), as approximately 25,000 cases of multidrug-resistant (MDR, resistant to at least isoniazid (INH) and rifampicin (RIF)) or rifampicin-resistant (RR) TB emerged in 2023. However, only about 40% of these cases have been laboratory-confirmed due to limited detection capacity [1]. Compared with drug-sensitive TB cases, DR-TB treatment is more complicated, long-lasting, and expensive, associated with increased adverse effects and a lower cure rate. One of the most economical and feasible measurements to curb DR-TB prevalence is to rapidly and accurately diagnose these patients.

Molecular diagnostic technology has been booming in recent years, providing a powerful tool in DR-TB detection. Currently, Xpert MTB/RIF (Xpert, Cepheid, USA) is considered as the most popular molecular diagnostics worldwide. Xpert assay is a rapid diagnostic technology recommended by the World Health Organization (WHO), and can quickly detect rifampicin (RIF) resistance in about two hours after sample collection [2, 3]. However, identifying RIF resistance only is insufficient to develop an appropriate treatment regimen. Due to the lack of a method to timely identify isoniazid (INH) susceptibility and the long turn-around time of phenotypic drug susceptibility testing (pDST), it could take months for an MDR-TB to be diagnosed. This regrettable fact severely reduces the value of Xpert as a rapid molecular test. Molecular tests targeting other drugs have become available in recent years. Other molecular tests, such as the MeltPro TB assay (Zeesan Biotech, Xiamen, China), utilize melting curve analysis to detect resistance to key first- and some second-line anti-TB drugs [4]. However, its limited sensitivity with clinical specimens has restricted its widespread use [5]. Additional molecular diagnostics capable of detecting INH resistance, such as Cepheid’s Xpert MTB/XDR, Abbott’s RealTime MTB RIF/INH, Becton Dickinson’s BD Max MDR-TB, and Roche Molecular Diagnostics’ cobas MTB-RIF/INH, are not yet universally applied globally, including China. The Line Probe assay (LPA) (Hain Lifescience, Germany) was the first registered genotypic drug susceptibility test for TB in China. However, due to its complex procedure and high risk of contamination, it has gradually been phased out with the emergence of newer methods.

To optimize the rapid diagnostic capabilities of Xpert, WHO recommends using RR as a marker of MDR and suggests that RR patients should be promptly begin treatment according to the MDR chemotherapy regimen [6]. This strategy addresses the delays in treatment that result from the incapability of INH-resistance detection, thereby reducing the risk of MDR-TB transmission. However, data from Zhejiang Province of China revealed that only 77.7% of RR patients were confirmed to have MDR-TB [7]. A meta-analysis conducted in China assessing the reliability of RR as an indicator of MDR-TB found an overall reliability of 77%, with considerable regional variation, ranging from 57 to 95% [8]. These findings negate using RR to predict MDR-TB in China.

Furthermore, INH resistance is estimated to be the most common form of drug resistance, affecting around 8% of all TB patients [9]. However, the widespread use of the Xpert assay somewhat weakened INH resistance detection. A large meta-analysis found that INH resistance is significantly associated with higher risks of treatment failure (incidence rate ratio [IRR], 10.9), relapse (IRR, 1.8), and acquisition of additional drug resistance (IRR, 5.1) [10]. These findings underscore a urgent need for rapid molecular tests that capable of simultaneously detecting resistance to RIF and INH.

This study introduces a novel molecular test kit, Innowave MTB/RIF/INH (InnowaveDX) by Suzhou Chuanglan Ltd., China, which can detect INH and RIF resistance simultaneously within 3 h after sample collection, allowing for an MDR-TB diagnosis on the first day of a hospital visit. InnowaveDX is also designed for TB diagnosis purposes by incorporating *IS6110* as a detection target, which theoretically offers high sensitivity due to the presence of multiple copies of this insertion sequence in the *Mycobacterium tuberculosis* (Mtb) genome. Additionally, it includes *rpoB* for RIF resistance detection, and *katG*, *inhA*, and *ahpC* for INH resistance detection. Unlike the Xpert assay, InnowaveDX requires a separate DNA extraction process involving a few simple steps that take about 30 min in total; therefore we defined it as a near point-of-care test (POCT). Notably, the DNA amplification and detection processes could be performed on an open platform of PCR equipment, making it a versatile option for various laboratory setups.

## Materials and methods

### Study design and participants

**Fig. 1:**
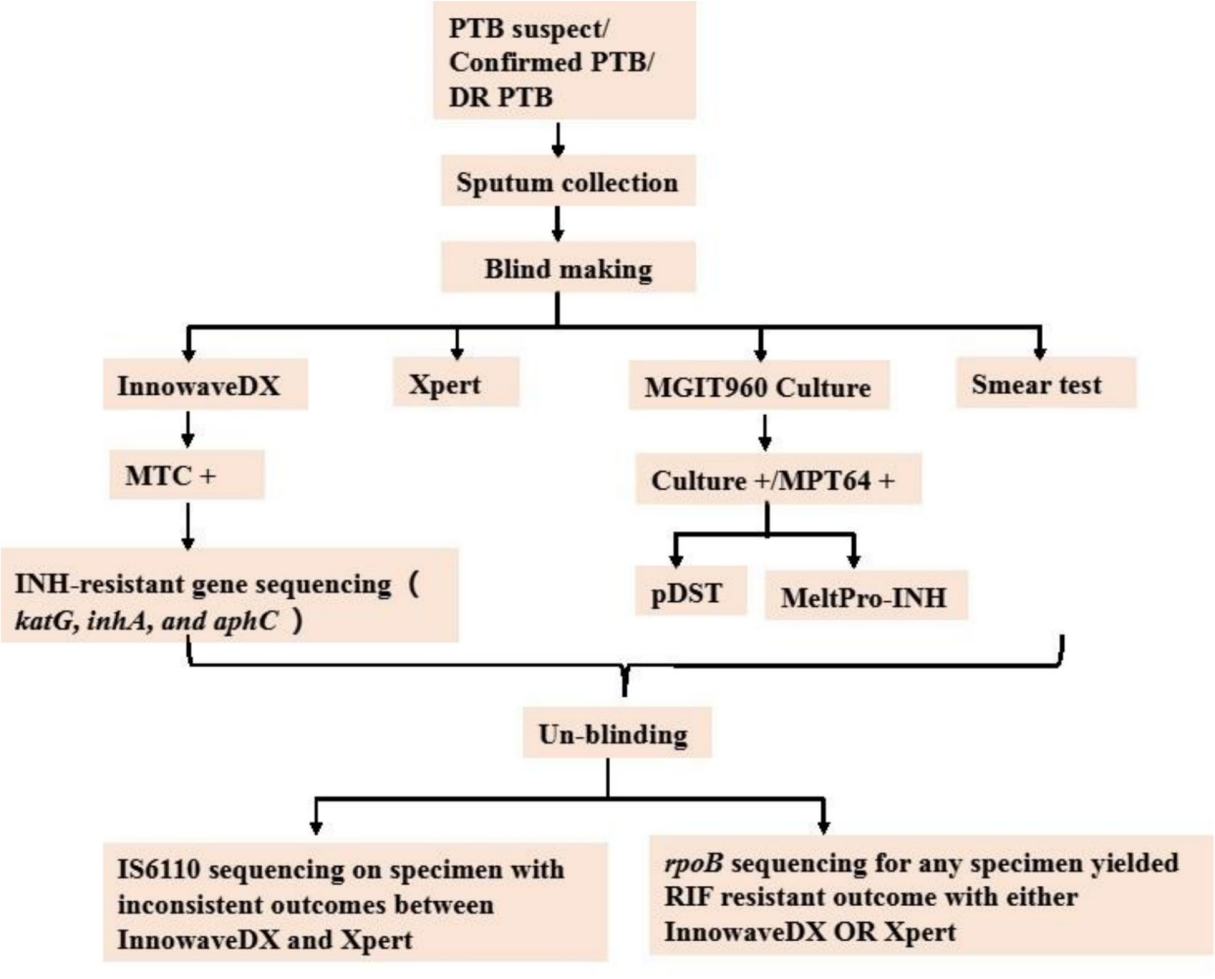
Flowchart of the on-site evaluation of InnowaveDX

Participants were enrolled prospectively and consecutively from February to December 2023 in 3 tertiary hospitals located in three different provinces of China: Beijing Chest Hospital (Beijing), Hangzhou Red-cross Hospital (Zhejiang province), and The Sixth People’s Hospital of Zhengzhou (Henan province). Based on the purpose of the test, two different recruitment criteria were applied. (1) To define the performance of InnowaveDX in PTB diagnosis, TB suspects were consecutively recruited. According to the WHO standard criteria, a TB suspect was defined as a case presenting symptoms and X-ray examination signs suggestive of pulmonary TB [11]. (2) To evaluate the drug- resistance detection performance, confirmed pulmonary TB patients were recruited, regardless of their anti-TB treatment status. Priority was given to PTB patients who were smear-positive and/or had positive molecular test results with drug-resistant outcomes from routine laboratory diagnoses. Each sputum specimen, with a volume of more than 5 mL, underwent simultaneous testing with smear tests, culture, Xpert, and InnowaveDX. For any specimen that was culture-positive and confirmed as *M. tuberculosis* complex (MTBC) via MPT64 antigen testing, pDST and the MeltPro assay, which targets INH resistance through *katG* and *inhA* genes, were performed. Sputum specimens testing positive for MTBC by InnowaveDX were further analyzed for INH resistance gene sequencing, which included *katG*, *inhA*, and *ahp*C. For specimens identified as RIF-resistant by either InnowaveDX or Xpert, the *rpoB* drug-resistant determination region (RRDR) was sequenced. To avoid bias, each test was assigned a separate blind code, and patient information along with the outcomes of other tests was concealed from the operators. The flowchart for this on-site evaluation study is shown in Fig. 1.

### Patient categories

Definite pulmonary TB is defined as culture-confirmed TB, where a positive culture result and the MPT64 antigen test indicate MTBC. Smear positivity alone is not considered direct evidence of confirmed TB, as many of them are later identified as non-tuberculous mycobacteria (NTM). Probable TB refers to cases where TB culture results are negative, but the patient is clinically diagnosed with TB based on clinical findings, radiologic imaging, and a positive response to tentative anti-TB treatment. Non-TB patients are those diagnosed with other diseases or where laboratory tests did not indicate TB, and who showed clinical improvement without receiving anti-TB treatment.

### Smear and culture

Direct smear was prepared and stained with auramine and then examined using light-emitting diode microscopy. Broth based liquid culture using the MGIT 960 system (BD Diagnostic Systems, NJ, USA) was conducted according to the manufacturer’s instructions. For all isolates, MPT64 antigen testing (Kaibili Ltd., China) was performed to confirm the presence of MTBC.

### Xpert MTB/RIF assay

The Xpert assay was performed on 1 mL of the sputum according to the manufacturer’s instructions by the GeneXpert instrument (Cepheid Inc., Sunnyvale, CA, USA).

### InnowaveDX MTB/RIF/INH assay

The detection process followed the instructions of the manufacturer. One milliliter of sputum was mixed with 6 mL of processing buffer and placed in a sonicator for 5 min to ensure full homogenization. The specimen was then transferred to a designated slot pre-loaded with lyophilized reagents and processed in the automatic DNA extraction equipment (Innovo-QS, Suzhou Chuanglan Ltd., China) for 25 min. The supernatant was subsequently added to a reaction tube and loaded into the SLAN-99S Real-time PCR system (Hongshi Tech., China) for a 2-h amplification. Following amplification, the software automatically analyzed the results for MTBC detection and mutations associated with RIF- and INH-resistance. Human β-actin gene is set up as the internal control for DNA extraction and amplification monitoring.

### Gene sequencing for IS6110, rpoB, katG, inhA, aphC

Sputum was processed for DNA sequencing of *IS6110* to verify TB DNA existence for diagnosis purposes; RRDR of *rpoB* gene to identify mutations associated with RIF resistance; genes of *katG, inhA,* and *aphC* that covered the major locus related with INH resistance. Gene amplification was conducted using a previously published method [12], and the sequencing was performed by Shanghai Sangon Company (China). DNA sequences were analyzed and compared with the reference sequence of the Mtb strain H37Rv using Lasergene™ software version 7.1.0 (DNASTAR, USA).

### Drug susceptibility testing

Phenotypic DST was performed on the isolates using the BACTEC MGIT 960 system (BD Diagnostic Systems, USA), with a critical concentration of 1 mg/L for RIF and 0.2 mg/L for INH, respectively.

### MeltPro TB assay

MeltPro TB assay was performed following the manufacturer’s instructions [4]. Crude DNA was extracted from the isolate using a paramagnetic particle method on an automatic DNA extraction machine (Zeesan Biotech, China). Amplification and melting curve analysis were conducted on the SLAN-99S Real-time PCR system (Hongshi Tech., China), and the Tm values were determined by identifying the peaks in the melting curves. The genotype of the sequence was judged according to the Tm value differences.

### Statistical analyses

McNemar’s test was used to compare the sensitivity and specificity of PTB diagnosis and drug-resistance detection between methods. Statistical analysis was conducted using SPSS version 19.0, with differences considered statistically significant at P < 0.05.

## Results

### Patient characteristics

A total of 1,101 participants were enrolled across the three sites, with 48 cases excluded due to undetermined diagnosis. Among the 1,053 eligible patients, 767 were diagnosed with pulmonary tuberculosis (PTB), including 365 with definite PTB and 402 with probable PTB, while 286 were non-TB cases. The non-TB cases included pneumonia (178), non-tuberculous mycobacterial (NTM) pulmonary disease (52), lung cancer (18), chronic obstructive pulmonary disease (22), and other conditions (16). All participants were HIV-negative. The median age was 57 years, ranging from 13 to 91 years, and women comprised approximately one-third (29.44%) of the participants.

### Performance of InnowaveDX and Xpert assay in PTB diagnosis

Among the 767 PTB cases, InnowaveDX demonstrated higher sensitivity (74.97%, 575/767) in MTB detection compared to the Xpert assay (68.18%, 523/767) (p = 0.003, χ^2^ = 8.664). There were 365 culture-positive PTB cases, including 276 cases that yielded smear-positive outcome as well. In cases of definite PTB, InnowaveDX and Xpert assay showed similar sensitivity [99.45% (363/365) vs. 97.8% (357/365), P = 0.056, χ^2^ = 3.65]. Further stratified analysis indicated that both methods detected nearly all of the 276 smear-positive and culture-positive cases, with InnowaveDX identifying 275 cases and Xpert 273 cases. Among the 89 smear-negative and culture-positive cases, InnowaveDX detected slightly more positive cases than Xpert (88 vs. 84), but the difference was not statistically significant (P = 0.097, χ^2^ = 2.760). In probable PTB cases, InnowaveDX exhibited higher sensitivity than the Xpert assay [52.73% (212/402) vs. 41.29% (166/402); p = 0.001, χ^2^ = 10.565]. In the 286 non-TB cases, InnowaveDX had similar specificity to the Xpert assay (97.9%, 280/286 vs. 99.3%, 284/286; P = 0.154, χ^2^ = 2.028). Two patients with a history of TB showed positive results on both InnowaveDX and the Xpert assay, with the presence of TB DNA confirmed by *IS6110* sequencing of the sputum samples.

### Performance of InnowaveDX in predicting RIF resistance

Among the PTB patients, 522 cases yielded positive results with both InnowaveDX and the Xpert assay, although 3 cases had indeterminate RIF outcomes with the Xpert assay. Of the 519 cases with conclusive results, 330 were RIF-sensitive as predicted by both methods, and 175 were RIF-resistant by both. The overall concordance rate for RIF susceptibility prediction between these methods was 97.3% (505/519). In the 14 discordant cases, 8 were resistant according to InnowaveDX but sensitive according to the Xpert assay. Among these, 4 were culture-positive and RIF-sensitive by pDST; *rpoB* mutations in RRDR were identified in 4 out of the 6 cases by sequencing. The remaining 6 discordant cases with InnowaveDX-sensitive but Xpert-resistant outcome, and 2of them were culture-positive and RIF-resistant by pDST, and all 6 cases had *rpoB* mutations identified by sequencing.

**Fig. 2:**
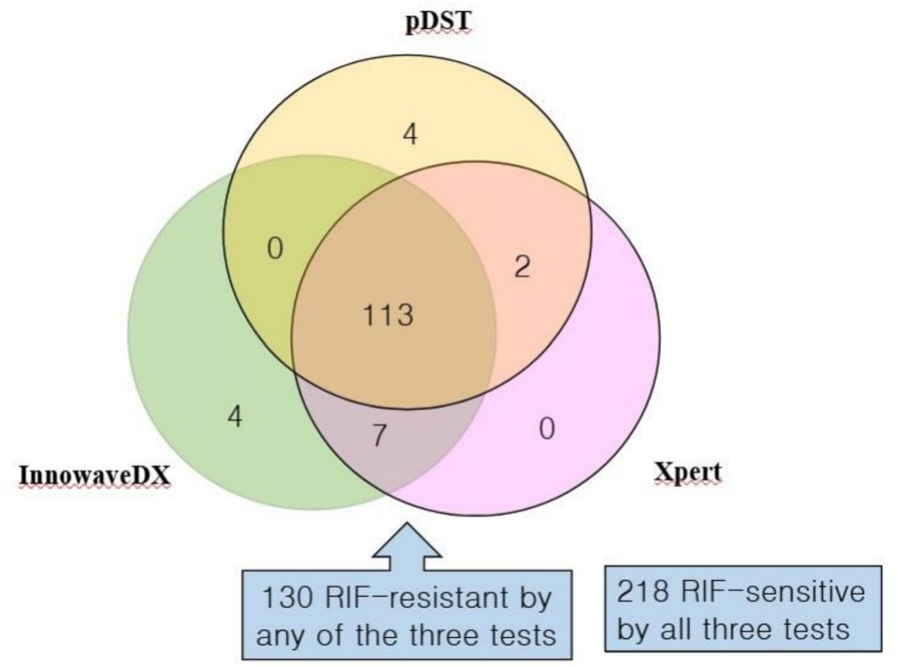
The outcomes of the 348 PTB patients with interpretable RIF-susceptibility data

A total of 348 cases had interpretable RIF-resistance detection outcomes from InnowaveDX, Xpert, and pDST. Of these, 218 cases were RIF-sensitive, and 113 were RIF-resistant according to all three methods. The overall concordance rate across the three methods was 95.11% (331/348) (Fig. 2). pDST and InnowaveDX uniquely identified 4 RIF-resistant cases, but none for Xpert assay.

In 190 cases who successful sequenced *rpoB* on sputum specimens, 182 harbored mutations in RRDR, and InnowaveDX detected 96.7% (176/182) of them. Except for three negative results, Xpert detected 98.88% (177/179) of these RIF-resistant cases indicated by sequencing. Similarly, when compared with pDST, among the 121 cases that showed RIF-resistant outcomes by pDST, InnowaveDX failed in one case, and detected 96.64% (114/120) of the resistant cases. Xpert failed in two cases, detecting 95% (115/119). No significant difference was observed between the two molecular tests when using either *rpoB* sequencing or pDST as the reference method (p = 0.160, χ^2^ = 1.978; p = 0.527, χ^2^ = 0.40).

### Performance of InnowaveDX in predicting INH resistance

**Fig. 3:**
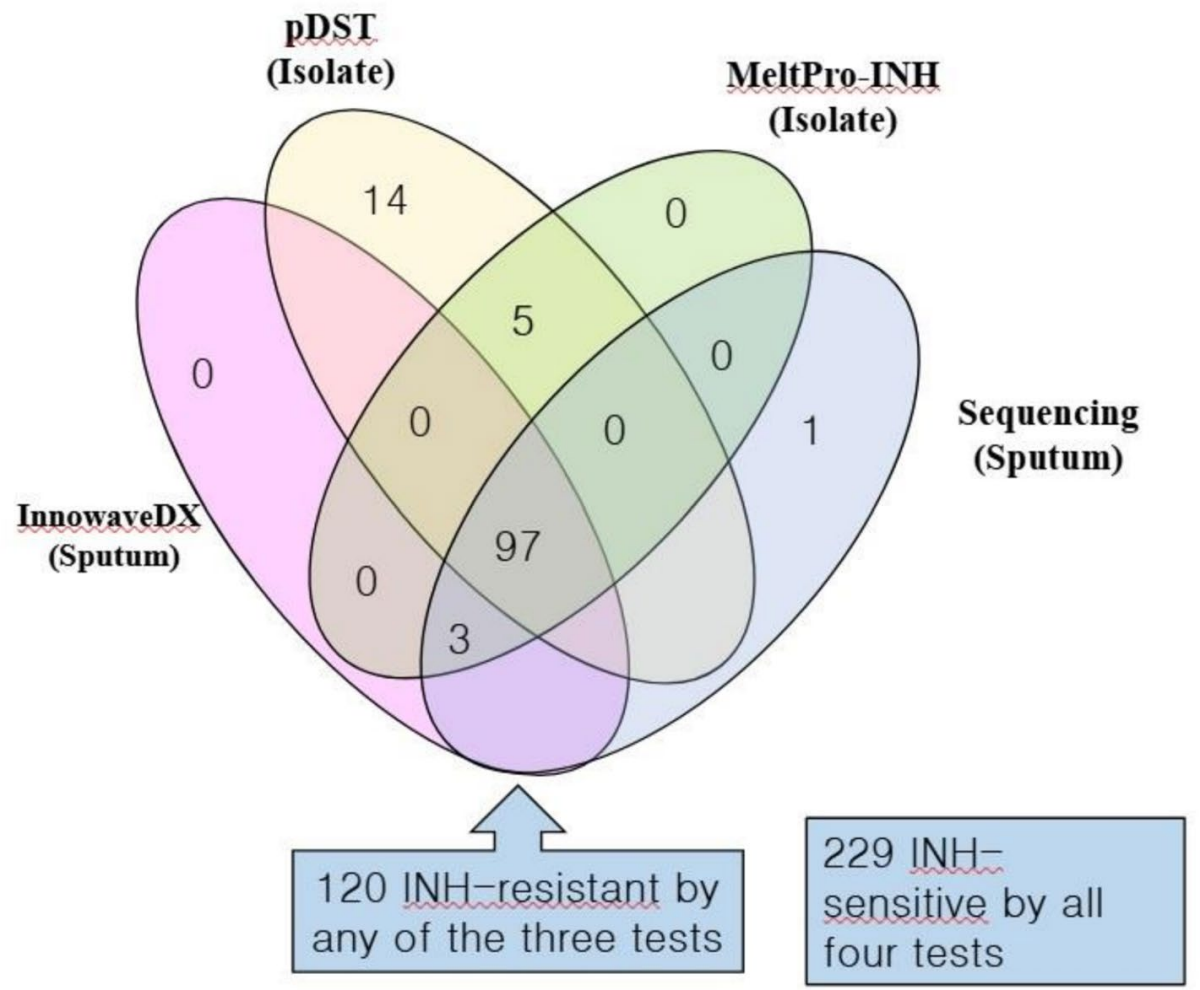
The outcomes of the 349 PTB patients with interpretable INH-susceptibility data

InnowaveDX identified 83.05% (98/118) of the INH-resistant cases predicted by pDST, 95.45% (105/110) by MeltPro-INH performed on isolates, and 99.35% (154/155) by *katG*, *inhA*, and *aph*C sequencing on sputum samples. Additionally, 349 cases yielded interpretable outcomes for concurrent testing by InnowaveDX, Xpert assay, pDST, and MeltPro-INH. Of these, 229 cases were predicted as INH-sensitive by all four methods, and 97 as INH-resistant. The overall concordance rate among these four methods was 93.4% (326/349) (Fig. 3). Most discordant results involved cases identified as INH-resistant only by pDST.

Among the 390 RIF-sensitive cases detected by InnowaveDX, 36 (9.23%) were identified as INH-resistant by InnowaveDX. These INH-resistant cases were confirmed by sputum DNA sequencing (36/36), pDST (17/18), and MeltPro-INH (20/20) performed on isolates.

### Performance of InnowaveDX in predicting MDR

**Fig. 4:**
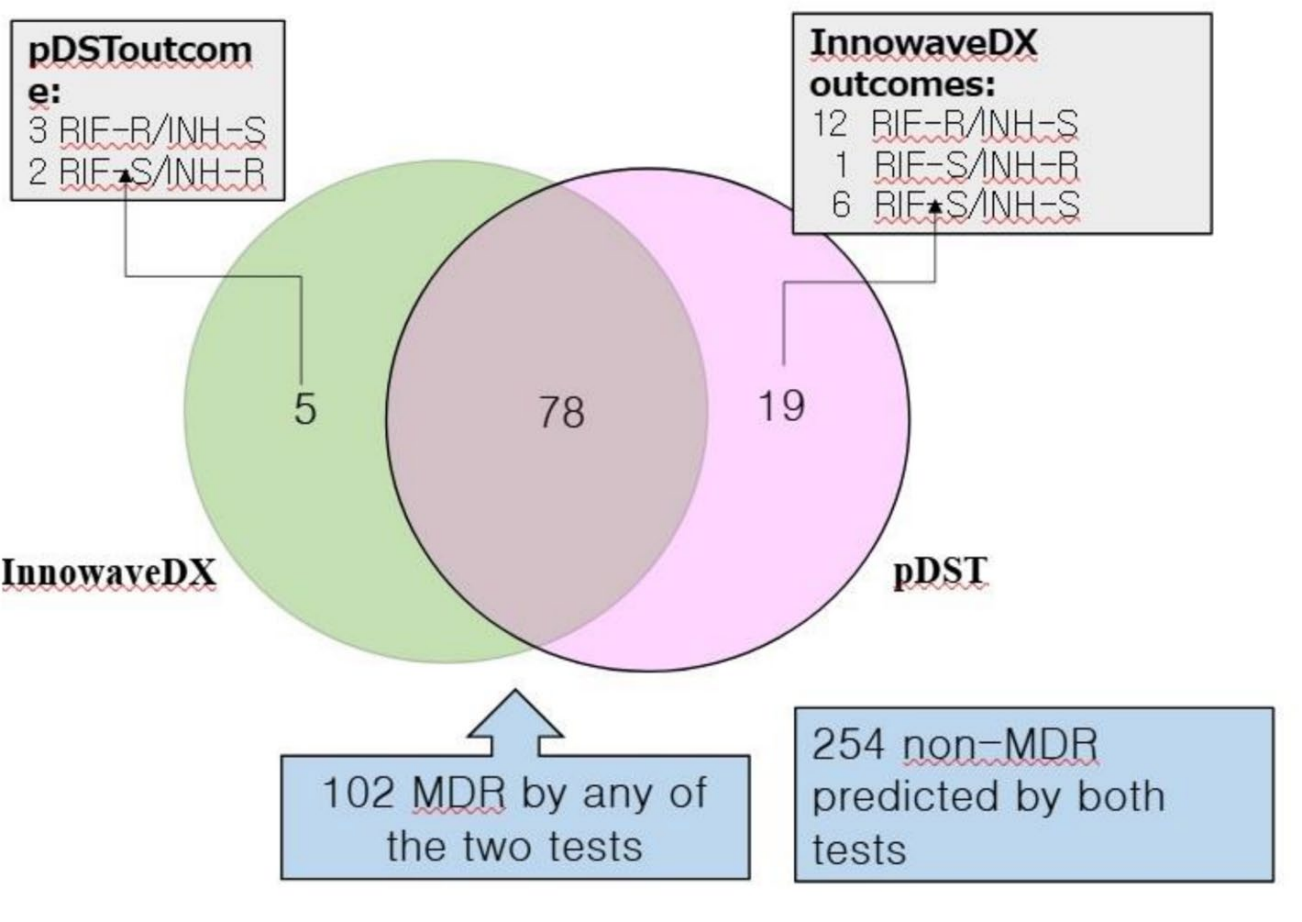
The outcomes of 356 PTB patients with interpretable pDST and InnowaveDX outcomes for MDR prediction

Among the PTB patients, 356 cases yielded interpretable outcomes from both InnowaveDX and pDST for INH and RIF resistance detection. A total of 78 cases were identified as MDR-TB, and 254 as non-MDR-TB by both methods. The overall concordance rate for MDR-TB diagnosis between the two methods was 93.25% (332/356). More MDR cases were predicted by pDST than by InnowaveDX, with 12 out of the 24 discordant cases showing RIF-resistant but INH-sensitive results according to InnowaveDX, while pDST classified them as MDR-TB (Fig. 4).

### The accuracy of MDR-TB prediction by RR diagnosed with different methods

The reliability of using RR to predict MDR-TB, based on different methods for determining INH susceptibility, was evaluated (Table 1). The prediction accuracy ranged from 64.1 to 80.5%. Regardless of the RR prediction method, referring to INH resistance as determined by pDST provided the highest reliability for MDR prediction. The highest accuracy (80.5%) was achieved when both RR and INH susceptibility were assessed by pDST.

**Table 1:** The MDR ratio among RR patients detected by different methods

RR detection method (n)	INH-R rates among successful cases			
	InnowaveDX (Sputum)	Sanger sequencing (Sputum)	pDST (Isolate)	MeltPro-INH (Isolate)
InnowaveDX(186)	64.5% (120/186)*	64.1% (118/184)*	75.2% (94/125)	69.5% (89/128)
Xpert (181)	65.2% (118/181)*	64.8% (116/179)*	77.0% (94/122)	70.6% (89/126)
pDST(128)	66.9% (81/121)*	66.7% (80/120)*	80.5% (103/128)	71.7% (86/120)
Sanger Seq (182)	64.8% (118/182)*	64.4% (116/180)*	77.7% (94/121)	71.2% (89/125)

^*^Compared with INH resistance predicted by pDST, P < 0.05

## Discussion

Real-world data from various settings indicate that rifampicin resistance (RR) does not always reliably predict multidrug-resistant TB (MDR-TB) [13, 14]. Given the critical role of isoniazid (INH) in TB treatment, it is not advisable to discontinue INH solely based on this MDR prediction [15, 16]. Therefore, a rapid molecular test capable of accurately detecting both RIF and INH resistance promptly would be valuable to overcome the limitations of the above prediction strategy. In recent years, next-generation sequencing (NGS) has emerged as a powerful tool for detecting drug resistance gene mutations. However, NGS is still not ready for widespread clinical use due to the high costs of equipment and reagents, the complexity of the procedure, and the need for specialized data interpretation. Furthermore, lower sensitivity of this technique has been reported with clinical specimens [17]. In the future, point-of-care testing (POCT) for detecting drug resistance in major anti-TB drugs will likely remain the primary approach in clinical practice, especially in peripheral clinical centers.

In this multiple-centered on-site evaluation, InnowaveDX outperformed GeneXpert MTB/RIF in PTB diagnosis with higher sensitivity (74.97% V.S 68.18%; p = 0.003), with no compromise in specificity (97.9% and 99.3%). The improved sensitivity of InnowaveDX was especially notable in culture-negative patients, where it achieved 10% greater sensitivity than the Xpert assay [52.73% vs. 41.29% (166/402); p = 0.001]. This increased sensitivity may be attributed to the difference in targeted genes between the two tests. It is well known that *IS6110*, the target gene in InnowaveDX, often has multiple copies in the genome of clinical Mtb isolates, whereas *rpoB*, the target gene in GeneXpert, is a single-copy gene. This premise of multiple gene copies is also the basis for the increased sensitivity observed in Xpert Ultra, besides *rpoB* gene, it includes *IS6110* and *IS1061* as detection targets as well [18]. Additionally, although the DNA extraction process a few more steps, it makes better homogenization of sputum and may acquire better DNA yields, which could also be attributed to the higher sensitivity of InnowaveDX.

InnowaveDX and Xpert showed a very high concordance rate in RIF susceptibility prediction (97.3%, 505/519). Furthermore, when using *rpoB* sequencing on sputum or pDST with clinical isolates as reference methods, both InnowaveDX and Xpert achieved good sensitivities (> 95%). InnowaveDX also demonstrated good performance in predicting INH susceptibility. When using pDST and Sanger sequencing as reference standards, the sensitivity of InnowaveDX for detecting INH resistance was 83.3% and 98.1%, while the specificity was 99.2% and 100%, respectively. The slightly lower detection rate of INH resistance using pDST as the reference method is a common occurrence in INH molecular testing, as the molecular mechanisms underlying INH resistance are not yet fully understood [19]. Even though InnowaveDX includes *katG, inhA,* and *aphC* as the gene targets, there is still an over 10% gap that the phenotypic INH resistance could not be explained by mutations in these canonical genes [20]. Consequently, higher sensitivity was achieved when using Sanger sequencing on sputum samples targeting these three genes (99.35%) or MeltPro-INH, which detects *katG* and *inhA* mutations, as the reference standard (95.45%). Furthermore, variations in the test samples (sputum or isolates) and potential heteroresistance due to the presence of both drug-sensitive and drug-resistant bacterial populations could contribute to inconsistencies between tests [21]. Notably, 9.23% of RIF-sensitive cases predicted by InnowaveDX were INH-resistant, underscoring the need for a timely and culture-independent test for INH resistance to enable more precise treatment regimens. Failure to detect these INH-resistant but RIF-sensitive cases may accelerate the development of MDR-TB if RIF/INH regimens are used, as effective RIF monotherapy could drive the evolution to MDR.

InnowaveDX demonstrated high concordance with pDST for MDR prediction (93.25%, 332/356). It is not surprising that pDST predicted more MDR cases than InnowaveDX, as it identified more INH-resistant cases (Fig. 4). Given the enriched data collected, we conducted an intensive analysis to evaluate the accuracy of RR as a predictor of MDR-TB (Table 1). Regardless of the resistance detection method used, the strategy of using RR to predict MDR was only moderately reliable (64.5%–80%), consistent with previous reports from China [8]. Although pDST-predicted RR showed slightly better accuracy, the long turnaround time of pDST limits its practicality for timely MDR prediction. An approximate 70% accuracy for MDR prediction using rapid molecular RR detection suggests that applying this strategy indiscriminately may result in many INH-sensitive patients losing the opportunity to benefit from this effective, inexpensive, and safe drug. This is unfortunate for patients who are already resistant to RIF, a key drug in the standard treatment regimen. Therefore, rapid molecular tests capable of detecting both INH and RIF resistance simultaneously would help to solve this dilemma. InnowaveDX can identify INH and RIF resistance within three hours of specimen receipt, with a few manual sample processing steps. Additionally, its nucleic acid amplification and detection platform is compatible with various PCR equipment brands. This flexibility is particularly valuable, as PCR machines were widely deployed during the COVID-19 pandemic around the country, and making good use of these devices in different levels of hospitals is a pertinent topic in the post-pandemic era. Moreover, the sample processing buffer contains high concentrations of guanidine and alcohol, enabling rapid and potent bacilli inactivation. We clarified the sterilization efficacy of the sample processing method with 10 Xpert-positive sputa and *M. tuberculosis* H37Rv reference strain at different concentrations, and found that the sample processing buffer alone or integrated with the sonication step, can kill bacilli efficiently (data not shown). This result demonstrates that the sample processing method presents minimal biohazard risk. This feature makes the method suitable for use in peripheral laboratories with limited facilities.

This study has several limitations. Firstly, as a trifunctional test that includes drug resistance detection, confirmed TB cases and PTB cases with a high risk of drug resistance were more likely to be enrolled. Therefore, the outcomes of this study is not objectively represent the epidemiological features of PTB and DR-TB patients in China. Secondly, although a smear test was performed for each enrolled patient, we did not conduct an intensive analysis for smear-positive cases. As NTM was frequently isolated in all the participating hospitals, and many smear-positive cases did not yield culture-positive results to confirm their species. Thirdly, there are two versions of the InnowaveDX test: one fully automated on a dedicated equipment platform, similar to the Xpert assay, and another that is open to PCR amplification platforms but requires several manual processing steps. This study only evaluated the version requiring more manual handling, while the performance of the fully automated version warrants separate evaluation.

In conclusion, InnowaveDX has proven to be an easy-to-use, rapid, and highly sensitive molecular test for PTB diagnosis. This technique can also identify INH and RIF resistance within 3 h, facilitating MDR-TB diagnosis on the first day of a hospital admission. Notably, nucleic acid amplification and detection can be performed on general PCR equipment, making its application more accessible.

## Data Availability

No datasets were generated or analysed during the current study.

## Abbreviations

POCT: Point-of-care test
RRDR: Rifampicin-resistance determining region
KatG: Catalase-peroxidase
InhA: NADH-dependent enoyl-[acyl-carrier-protein] reductase
AhpC: Alkyl hydroperoxide reductase C protein
